# Análise do Conteúdo de Aplicativos Móveis Brasileiros Voltados ao Controle da Pressão Arterial: Uma Busca Sistemática

**DOI:** 10.36660/abc.20240339

**Published:** 2025-02-05

**Authors:** Eduardo Braghini Johann, Marcelo Baggio, Nicolle Abreu Pauli, Artur Dilli, Rafael Teixeira Ilkiu, Raphael Mendes Ritti-Dias, Aline Mendes Gerage

**Affiliations:** 1 Grupo de pesquisa em exercício clínico Universidade Federal de Santa Catarina Florianópolis SC Brasil Grupo de pesquisa em exercício clínico (GPEC) – Universidade Federal de Santa Catarina, Florianópolis, SC – Brasil; 2 Programa de Pós-Graduação em Educação Física Centro de Desportos Universidade Federal de Santa Catarina Florianópolis SC Brasil Programa de Pós-Graduação em Educação Física, Centro de Desportos – Universidade Federal de Santa Catarina, Florianópolis, SC – Brasil; 3 Programa de Pós-Graduação em Ciências da Reabilitação Universidade Nove de Julho São Paulo SP Brasil Programa de Pós-Graduação em Ciências da Reabilitação – Universidade Nove de Julho, São Paulo, SP – Brasil

**Keywords:** Saúde Digital, Pressão Arterial, Hipertensão, Telemedicina, Assistência Médica

## Abstract

**Fundamento:**

Vários Aplicativos (apps) foram desenvolvidos para auxiliar no manejo da hipertensão, mas pouco se sabe sobre a qualidade desses apps no cenário brasileiro,

**Objetivos:**

Identificar e analisar a qualidade dos apps que visam o controle da pressão arterial (PA) disponíveis em português.

**Métodos:**

Uma busca sistemática foi realizada nas lojas virtuais de apps do Brasil nos sistemas operacionais Android e iOS de novembro de 2021 a março de 2022, com uma atualização em março de 2024. A busca baseou-se nos padrões de revisão sistemática (PRISMA), utilizando palavras-chaves pré-definidas, incluindo apps em português, gratuitos e disponíveis para uso durante a busca. Três revisores independentes analisaram os apps usando a escala de cinco pontos MARS (Mobile App Rating Scale), e também quanto à presença de ferramentas e conteúdos sobre controle da hipertensão.

**Resultados:**

Cinquenta e seis apps preencheram os critérios para extração dos dados. A ferramenta mais prevalente foi o registro dos valores da PA (98%). A ferramenta para adesão aos medicamentos e lembretes para seu uso esteve presente em somente 29% e 34% dos apps, respectivamente. A média do escore MARS foi 3,4 ± 0,74 e 3,1 ± 0,61 para os apps do sistema Android e do sistema iOS, respectivamente. O item mais bem avaliado foi “funcionalidade” tanto para os apps Android como iOS.

**Conclusão:**

O presente estudo identificou vários apps de qualidade aceitável direcionados ao monitoramento da PA. Contudo, a maioria deles não incluíam fatores importantes relacionados ao controle da doença particularmente em relação à adesão ao tratamento, atividade física e presença de comorbidades.

## Graphical abstract

**Figura Central:**
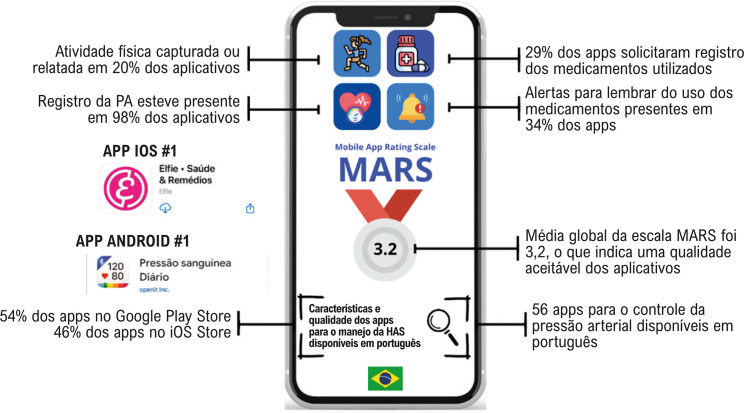
Análise do Conteúdo de Aplicativos Móveis Brasileiros Voltados ao Controle da Pressão Arterial: Uma Busca Sistemática

*HAS: hipertensão arterial sistêmica.*

## Introdução

A hipertensão arterial sistêmica (HAS), um dos principais fatores de risco para doenças cardiovasculares, é uma condição prevalente na população adulta e associada a altos custos para a saúde pública.^1 , 2^ Apesar da comprovada eficácia dos medicamentos e das abordagens ao estilo de vida, a maioria dos pacientes hipertensos não têm a doença sob controle.^3 , 4^

Um rastreamento global conduzido em 54 países revelou que as taxas de conhecimento, tratamento e de controle da pressão arterial (PA) foram mais baixas que o esperado.^4^ Entre as principais razões para esse fato, destaca-se a baixa adesão ao tratamento, seja medicamentoso ou não.^5^ Assim, estratégias visando aumentar a adesão ao tratamento anti-hipertensivo continua um desafio contemporâneo. Isso ressalta a necessidade de se investir em novos métodos de intervenção no manejo da HAS, abordando os principais fatores relacionados ao controle da PA.

Os smartphones são os principais equipamentos utilizados para acessar a Internet,^6 , 7^ totalizando 249 milhões no Brasil. Assim, existe um interesse crescente em entender o potencial de aplicativos (apps) móveis direcionados a melhorar a saúde de várias populações.^8^ Para pacientes hipertensos, existem vários apps disponíveis com diversos recursos, como alarmes e lembretes para tomar a medicação, registro de níveis de PA, e conteúdo educativo.^9 , 10^ Contudo, uma busca sistemática indicou que a maioria dos apps na língua inglesa são de baixa qualidade.^11^ Não se sabe se resultados similares ocorrem com apps em português. Assim, o objetivo deste estudo é realizar uma busca sistemática para descrever as características e analisar a qualidade dos apps móveis direcionados ao manejo da PA, disponíveis m português.

A Figura Central resume os principais achados do estudo.

## Métodos

### Estratégia de busca e seleção

Realizou-se uma busca sistemática de apps móveis que visassem o manejo da PA nas lojas virtuais de apps do App Store e do Google Play Store, nos sistemas operacionais Android e iOS, respectivamente. A busca seguiu os padrões PRISMA de revisão sistemática,^12^ usando os termos “hipertensão”, “pressão arterial”, e “pressão alta”. A seleção dos apps baseou-se nas informações contidas na loja virtual, tais como o título, a descrição e a foto inicial do app. Foram selecionados para o presente estudo apps em português, gratuitos e disponíveis para uso durante a busca. Nos casos de apps que possuíam uma versão gratuita e uma versão premium, somente a primeira foi analisada; em caso de app duplicado, a escolha do sistema em analisar o app foi aleatória (proporção 1:1, em blocos de dois). Apps considerados irrelevantes ao tópico e apps em idioma diferente de português foram excluídos.

### Coleta de dados

A coleta de dados foi realizada por três pesquisadores independentes entre novembro de 2021 e março de 2022, com atualização em março de 2024 para verificação de novos apps. O primeiro investigador realizou a busca pelo sistema iOS usando um smartphone iPhone XR, o segundo realizou a busca pelo sistema Android usando um smartphone ASUS ZenFone Max Shot e o terceiro por ambos os sistemas (usando um iPhone 8 Plus e um Moto G7 Play), a fim de comparar as informações coletadas pelos outros. Cada pesquisador extraiu os dados dos apps, realizando o *download* e os testando em seus aparelhos celulares.

### Análise dos apps

Os apps foram analisados em uma planilha quanto aos seguintes dados: registro de comorbidades, valores de PA, medicamentos em uso, e prática de Atividade Física (AF, medida ou capturada), presença de conteúdos educativos relacionados ao tema, e grupos/fóruns de apoio, e possibilidade de exportar os dados. Parcerias com instituições ou organizações em construção foram também analisadas.

### Classificação da qualidade do app

A escala MARS ( *Mobile App Rating Scale* ) foi usada para classificar os apps pela qualidade.^13^ O instrumento consiste em quatro sessões distintas; em cada sessão, “A” tem como objetivo avaliar engajamento, “B” visa avaliar a funcionalidade, “C” a estética, e “D” a informação. Essas sessões incluem 19 perguntas, cada uma com uma escala de cinco pontos – 1 inadequado, 2 baixo, 3 aceitável, 4 bom, 5 excelente. Embora o instrumento inclua uma sessão de avaliação subjetiva, representada pela letra “E” e mais quatro perguntas, essa sessão não foi utilizada no presente estudo. O escore total do app foi calculado pela média de todas as quatro sessões.

### Análise estatística

As variáveis categóricas foram apresentadas em frequência absoluta e relativa, e as variáveis contínuas foram apresentadas em média e desvio padrão. Para analisar a qualidade dos apps, foi analisado o escore médio em cada sessão. As características e a qualidade dos apps para o manejo da PA foram comparadas usando o teste do qui-quadrado para comparar proporções (variáveis categóricas) e o teste t de Student não pareado para comparar as médias, se a distribuição dos dados fosse normal (teste de Shapiro-Wilk). Foi adotado um nível de significância de 5%, e as análises foram realizadas no programa SPSS versão 20.0.

## Resultados

**Figura 1:**
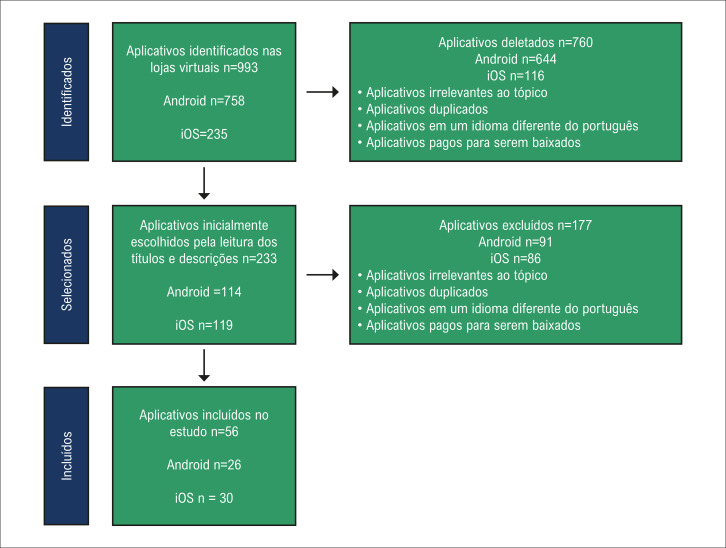
– Processo de seleção do aplicativo.

Um total de 993 apps foram identificados pela busca nos sistemas iOS e Android. Após a leitura dos títulos e descrições dos apps, 760 foram excluídos, restando 233 elegíveis para revisão. Depois que os apps foram baixados e filtrados quanto aos critérios, 56 preencheram os critérios para extração dos dados, 26 disponíveis no Android e 30 disponíveis no iOS. O processo completo encontra-se ilustrado na Figura 1 .

Um resumo das características dos apps é encontrado na Tabela 1 .

**Tabela 1:** – Resumo das ferramentas dos aplicativos avaliados

Variável	Total (n=56) n (%)	Android (n=26) n (%)	iOS (n=30) n (%)	Valor p
**Pressão arterial**				
Registro da pressão arterial	55 (98)	26 (100)	29 (97)	1,000
**Medicamentos**				
Medicamentos usados	16 (29)	9 (35)	7 (23)	0,388
Lembrete para tomar medicação	19 (34)	13 (50)	6 (20)	0,025
**Outros recursos**				
Registro de comorbidades	8 (14)	6 (23)	2 (7)	0,127
AF medida ou relatada	11 (20)	6 (23)	5 (17)	0,738
Educação em saúde	14 (25)	8 (31)	6 (20)	0,375
Possibilidade de exportar os dados	12 (21)	2 (8)	10 (33)	0,025
Fóruns	2 (4)	2 (8)	0 (0)	0,211
Parceria com organizações em saúde	3 (5)	3 (12)	0 (0)	0,094

*AF: atividade física.*

A ferramenta relacionada ao registro dos valores de PA esteve presente em 98% dos apps. Somente 29% dos apps solicitaram registro dos medicamentos utilizados, e somente 34% tinha alertas para lembrar o seu uso; isso foi mais comum nos apps de Android (50%) que nos de iOS (20%) (p=0,025). A opção de incluir comorbidades esteve presente em 14,0% dos apps. Somente 20% dos apps analisados apresentaram a possibilidade de capturar ou relatar AF, e 25% ofereceram conteúdo de educação em saúde para controle da HAS. Ainda, 21% dos apps possuíam a funcionalidade de exportar os dados, sendo mais frequentes nos apps iOS (33%) em comparação aos apps de Android (8%) (p=0,025). Somente 4% tinham fóruns de discussão e 5% tinham parceria com uma organização de saúde.

### Qualidade do aplicativo

O escore médio da qualidade dos apps foi 3,2±0,69. O escore mais alto foi para a funcionalidade dos aparelhos (3.7±0.73), enquanto a análise da informação recebeu o escore mais baixo (3,0±0,71). Ao comparar os escores dos apps do sistema Android com os escores dos apps do sistema iOS, observou-se uma diferença estatisticamente significativa em termos de informação (p=0,001), com melhor avaliação dos apps de Android (3,3±0,70 vs. 2,7±0,60) ( Tabela 2 ).

**Tabela 2:** – Média da qualidade do aplicativo segundo o instrumento MARS ( *Mobile App Rating Scale* ) de Stoyanov **1**

Subescalas MARS	Total	Android	iOS	Valor p
Engajamento	3,1 (,72)	3,2 (,74)	3,0 (,69)	0,211
Funcionalidade	3,7 (,73)	3,8 (,86)	3,5 (,58)	0,107
Estética	3,2 (,75)	3,3 (,79)	3,1 (,72)	0,361
Informação	3,0 (,71)	3,3 (,70)	2,7 (,60)	0,001
Escore MARS global*	3,2 (,69)	3,4 (,74)	3,1 (,61)	0,080

MARS: Mobile App Rating Scale; dados apresentados em média (desvio padrão). *Média das quatro subescalas objetivas.

### Aplicativos iOS com as médias mais altas

A Tabela 3 apresenta os cinco apps disponíveis na App Store mais bem avaliados pelo MARS.

**Tabela 3:** – Os cinco aplicativos iOS mais bem avaliados pelo *Mobile App Rating Scale* (MARS)

Características	Apps iOS				
	Mappa	AMPA	Blood Pressure Calendar	Blood Pressure Control	Elfie
**Escore MARS**	3,7	3,9	3,9	4,0	4,5
**Dados pessoais**					
**Pressão arterial**					
Registro dos valores	x	x	x	x	x
**Medicamentos**					
Medicamentos utilizados		x	x		x
Lembrete para o uso			x		
**Outros recursos**					
Registro de comorbidades		x		x	
AF capturada ou relatada		x	x		x
Educação em saúde	x			x	x
Possibilidade de exportar dados registrados		x	x		
Fóruns				x	
Parceria com organizações de saúde				x	

*AF: atividade física.*

Todos os apps do sistema iOS que receberam os escores MARS mais altos possuíam uma ferramenta para o registro das PAs. Dos cinco apps, três possuíam alguma forma de medir a quantidade de AF praticada pelo indivíduo com HAS.

### Aplicativos do Android com as maiores médias de MARS

A Tabela 4 apresenta os cinco apps disponíveis na loja virtual do sistema Android mais bem avaliados pelo MARS.

**Tabela 4:** – Os cinco aplicativos disponíveis no sistema Android mais bem avaliados pelo Mobile App Rating Scale (MARS)

Características	Android apps				
	Blood pressure - Blood journal	Heart monitor: blood pressure diary	Blood pressure (bpresso)	UCS Digital Health	Blood Pressure Diary
**Escore MARS**	4,0	4,0	4,0	4,1	4,5
**Dados pessoais**					
**Pressão arterial**					
Registro dos valores	x	x	x	x	x
**Medicamentos**					
Medicamentos utilizados	x		x		
Lembrete para o uso	x	x	x		
**Outros recursos**					
Registro de comorbidades		x	x	x	
AF capturada ou relatada			x	x	
Educação em saúde		x			
Possibilidade de exportar dados registrados					
Fóruns					
Parceria com organizações de saúde					

*AF: atividade física.*

Os cinco apps Android mais bem avaliados também apresentavam um formulário para registro da PA. Somente dois dos apps apresentaram alguma maneira de medir a AF nos indivíduos com HAS. A lista completa de apps avaliados e seus respectivos escores na escala MARS estão disponíveis no material suplementar. Além disso, na atualização de 2024, observou-se que nove apps foram suspensos, quatro no sistema Android e cinco no sistema iOS (destacados no material suplementar).

## Discussão

O objetivo do estudo foi identificar e analisar apps de dispositivos móveis que visavam o controle da PA, disponíveis em português nas duas principais plataformas – App Store e Google Play. Entre os principais achados, destaca-se que 55 apps (98%) apresentaram a opção de registrar os valores de PA, enquanto a ferramenta para adesão ao uso de medicamentos esteve presente em somente 16 apps (29%), e lembrete para o uso de medicamentos em 19 apps (34%). A relação entre o manejo da PA e a prática de AF não atingiu ¼ dos apps. A média de escore MARS global foi 3,2 ± 0,69, com informação, engajamento, estética e funcionalidade em ordem ascendente de escores na escala MARS.

O automonitoramento da PA parece indicar melhor controle dos valores de PA^14^ e estar associado a maior adesão medicamentosa.^15^ No entanto, poucos apps (21%) ofereceram a possibilidade de exportar os valores de PA e a opção de gerar relatórios com gráficos e tabelas, dificultando a comunicação entre o médico o paciente. Em um estudo prévio, cerca de metade dos apps (44%) analisados tinham opção de exportar os dados preenchidos pelos participantes, facilitando o compartilhamento dos dados com profissionais de saúde.^16^ Outro estudo relatou que a principal funcionalidade dos apps analisados foi o conteúdo educacional fornecido sobre a doença (59,1%), e a segunda foi a opção de automonitoramento da doença (53,2%).^17^Porém, os autores relataram que os conteúdos educacionais não foram verificados para analisar a qualidade da informação.

Embora a baixa adesão ao tratamento farmacológico por pacientes hipertensos já tenha sido bem demonstrada,^3 , 18 , 19^ os apps analisados pouco exploravam ferramentas para aumentar a adesão ao tratamento medicamentoso. Metanálises mostraram que os apps com ferramentas que visavam o controle do tratamento farmacológico tendiam a aumentar a adesão ao uso dos medicamentos prescritos.^8 , 20^ Portanto, os apps devem possuir um sistema de emissão de notificações, pelo preenchimento prévio dos horários dos medicamentos, para lembrar os usuários a tomarem seus medicamentos nos horários prescritos, facilitando a adesão aos medicamentos.^21 , 22^

Mudanças comportamentais são fundamentais no controle da PA, mas observamos que os apps brasileiros fazem pouca abordagem sobre essa questão em suas funcionalidades. No presente estudo, somente 14 (25%) apresentaram ferramentas cujo objetivo era educação em saúde dos usuários, e 12 (20%) dos apps continham ferramentas que facilitaram a adesão à AF. A promoção de mudança comportamental pelo mHealth (ou saúde móvel) tem sido investigada nos últimos anos, e parece direcionar os indivíduos para hábitos mais saudáveis.^23 - 25^Embora mais estudos sejam necessários para comprovar a efetividade das características para aumentar comportamentos positivos, é importante que apps explorem essas variáveis para encorajar comportamentos preventivos relacionados ao estilo de vida para o tratamento não farmacológico de indivíduos hipertensos.^3^

No contexto de uma avaliação mais qualitativa baseada na escala MARS, a variável funcionalidade foi a mais bem avaliada (3,7±0,73 de 5). As comparações entre os escores obtidos pelos apps Android e pelos apps iOS mostraram que, em todas as subescalas, os apps disponíveis no sistema Android obtiveram uma leve vantagem, com uma diferença estatisticamente significativa (p=0,001) somente para o aspecto da informação. Podemos observar similaridades com estudos prévios, nos quais a categoria objetiva com o escore MARS mais alto foi funcionalidade, com uma média global de 3,5 em 5,0. No entanto, diferentemente do presente estudo, em um estudo^11^ avaliando apps disponíveis nas lojas oficiais dos sistemas Android e iOS na Holanda relataram um escore MARS similar entre os apps do sistema Android e os apps do sistema iOS (2.63 vs 2.64).

Vale ainda mencionar que as fontes de informação dos apps incluídos neste estudo eram ausentes ou de baixa qualidade. Outros estudos também relataram baixa qualidade da fonte de informações presentes nos apps.^11 , 16 , 26 , 27^ Essa baixa qualidade e/ou falta de informação pode resultar no uso incorreto do app, uma vez que muitos usuários não familiares com o app podem inserir ou interpretar os dados de maneira errada. Assim, é importante que os apps foquem em interfaces simples, design atraente, textos claros e objetivos, para facilitar seu uso.^28^

Em nosso conhecimento, existe somente um estudo^29^ publicado que avaliou um app com foco no controle da PA, disponível na língua portuguesa. Esse estudo^29^ foi uma revisão narrativa, em que os autores realizaram uma busca somente do Google Play Store, incluindo um total de 267 apps. Nesse estudo, os autores listaram os apps por categoria, número e principais funcionalidades, e destacaram que a maioria dos apps tinham fins de entretenimento, com funcionalidades sem caráter científico, enfatizando a importância de um melhor controle em relação ao desenvolvimento e à disponibilidade de apps de mHealth.^29^ O presente estudo, então, oferece informações pertinentes sobre a qualidade, a funcionalidade, a estética, o engajamento e informações dos apps disponíveis na língua portuguesa. Ainda, nosso estudo descreve diferenças entre apps disponíveis no sistema Android e iOS.

Este estudo tem limitações, como a não inclusão de apps pagos ou a análise apenas das versões mais simples, o que limita nossas conclusões. Por se tratar de um estudo transversal, mudanças nas tendências da funcionalidade dos apps não são consideradas. Além disso, o fato de que apps duplicados em ambas as lojas (Android e iOS) tenham sido analisados em uma única plataforma, de maneira aleatória, deveria ser considerado na interpretação de nossos resultados.

No presente estudo, identificamos o número de apps dedicados ao controle da PA disponíveis no idioma português nos sistemas Android e iOS. A maioria dos apps apresentavam uma qualidade aceitável, mas, em geral, não eram abrangentes em termos dos fatores relacionados ao controle da PA. Não existem estudos avaliando a eficácia desses apps, destacando a necessidade de realização de estudos para esse fim. Ainda, nossos resultados enfatizam a importância de se desenvolver apps que promovam maior adesão farmacológica e não farmacológica, e maior confiabilidade a profissionais de saúde recomendarem o seu uso, dado que o baixo conhecimento sobre apps é uma barreira para sua recomendação.^28^
