# Supplementary material for: Análise do Conteúdo de Aplicativos Móveis Brasileiros Voltados ao Controle da Pressão Arterial: Uma Busca Sistemática
Source: Arq Bras Cardiol. 2025 Feb 5;122(2):e20240339. [Article in Portuguese] doi: 10.36660/abc.20240339 (PMC11805575; doi:10.36660/abc.20240339)
Supplement: Translation

# Translation: Content Analysis of Brazilian Mobile Applications Targeting Blood Pressure Management: A Systematic Search

**DOI:** 10.36660/abc.20240339i

**Authors:** Eduardo Braghini Johann, Marcelo Baggio, Nicolle Abreu Pauli, Artur Dilli, Rafael Teixeira Ilkiu, Raphael Mendes Ritti-Dias, Aline Mendes Gerage

**Affiliations:** 1 Brazil Grupo de pesquisa em exercício clínico (GPEC) – Universidade Federal de Santa Catarina, Florianópolis, SC – Brazil; 2 Brazil Programa de Pós-Graduação em Educação Física, Centro de Desportos – Universidade Federal de Santa Catarina, Florianópolis, SC – Brazil; 3 Brazil Programa de Pós-Graduação em Ciências da Reabilitação – Universidade Nove de Julho, São Paulo, SP – Brazil

**Keywords:** Digital Health, Arterial Pressure, Hypertension, Telemedicine, Medical Assistance

## Abstract

**Background:**

Several mobile applications have been developed to assist in managing hypertension, but little is known about their quality in the Brazilian scenario.

**Objective:**

To identify and analyze the quality of the apps targeting blood pressure (BP) management available in Portuguese.

**Methods:**

A systematic search was carried out through Brazilian virtual app stores in the Android and iOS platforms from November 2021 to March 2022, with an update in March 2024. The search was based on the standards of systematic review (PRISMA), using predefined keywords, including apps in Portuguese, free and available for use during the search. Three independent reviewers analyzed the apps using the five-point Mobile App Rating Scale (MARS), and for the presence of tools/content related to the BP control.

**Results:**

Fifty-six applications met the criteria for data extraction. The most prevalent tool was the recording of BP values (98%). The tool for adherence to medication and reminders to their use was present in only 29% and 34% of the APPs, respectively. The mean overall MARS score was 3.4 ± 0.74 and 3.1 ± 0.61 for Android and iOS APPs, respectively, which classified then as acceptable. The best rated item was “functionality” for both Android and iOS apps.

**Conclusion:**

The present study identified numerous apps with acceptable quality aimed at monitoring BP. However, most of them did not include important factors related to disease control, particularly regarding treatment adherence, physical activity and presence of comorbidities.

## Introduction

Systemic arterial hypertension (SAH), one of the main risk factors for cardiovascular diseases, is a prevalent condition in adult population, associated with high costs for public health.^1 , 2^Despite the proven effectiveness of medication and lifestyle approaches, the majority of hypertensive patients do not have the disease under control.^3 , 4^

A global screening conducted in 54 countries found that awareness, treatment, and control rates of blood pressure (BP) were lower than expected.^4^Among the main reasons for that, low adherence to treatment, whether pharmacological and/or non-pharmacological, stands out.^5^Therefore, strategies to increase adherence to hypertensive treatment is still a contemporary challenge. This underscores the need to invest in new intervention methods to support the management of SAH, addressing the key factors related to BP control.

Smartphones are the most used equipment to access the internet,^6 , 7^totaling 249 million in use in Brazil. Therefore, there is a growing interest in understanding the potential of mobile applications directed to improve health in several populations.^8^For hypertensive patients, there are several available apps with different resources, such as alarms and reminders to take medication, recording of BP levels, and educational content.^9 , 10^However, a systematic search indicated that most of apps in English language are of low quality.^11^Whether similar results occur with apps in Portuguese, is unknown. Therefore, the objective of this systematic search is to describe the features and analyze the quality of the mobile APPs targeting BP management available in Portuguese.

The Central Illustration summarizes the main findings of the study.

## Methods

### Search and selection strategy

A systematic search of mobile APPs targeting BP management was carried out in the Brazilian virtual app stores of the App Store and Google Play Store, on the Android and iOS operating platforms, respectively. The search followed the PRISMA standards of systematic review,^12^using the terms “hypertension”, “blood pressure” and “high blood pressure. The selection was made based on the information contained in the virtual store, such as the app title, description and screen photos. Apps offered in Portuguese, free and available for use during the search were selected for the present study. As for Apps that had a free version and a premium version, only the first one was analyzed. When duplicated, the choice of system to analyze the App was random (1:1 proportion, in blocks of two). Apps irrelevant to the topic, as well as apps in a language other than Portuguese were excluded.

### Data collection

Data collection was carried out by three independent researchers from November 2021 to March 2022, with an update in March 2024 for new apps. The first investigator researched through the iOS platform using an iPhone XR smartphone, the second one through the Android platform using an ASUS ZenFone Max Shot smartphone and the third through both platforms (using an iPhone 8 Plus smartphone and a Moto G7 Play smartphone), to compare the information collected by the others. Each investigator extracted data from the apps, downloading and testing them on their respective cell phones.

### Application analysis

The apps were analyzed using a spreadsheet for the following data: recording of comorbidities, BP values, medications used, and practice of physical activity (PA) (either measured or captured), presence of health education content related to the topic and support groups/forums, and possibility of exporting the data. Partnerships with institutions or organizations in construction were also analyzed.

### Application’s quality rating

To classify the apps’ in terms of quality, the Mobile App Rating Scale (MARS) was used.^13^The instrument consists of four distinct sessions; in which section A is aimed at evaluating engagement, B for functionality, C for aesthetics and the last D, for information. These sessions include 19 questions, each with a five-point scale, with 1 inadequate, 2 poor, 3 acceptable, 4 good and 5 excellent. Although the instrument includes a subjective evaluation section, represented by the letter E with four more questions, for the present study, this section was not used. The APP total score was calculated by the average of all four sections.

### Statistical analysis

Categorical variables were presented in absolute and relative frequency, while continuous variables were shown using means and standard deviation. To analyze the quality of the apps, the average score in each section was analyzed. Characteristics and quality of the mobile apps targeting BP management were compared using the Chi-square test to compare proportions (categorical variables) and unpaired Student’s t-test to compare means, considering that normality was achieved (Shapiro Wilk test). A significance level of 5% was adopted. The statistical package used was SPSS, version 20.0.

## Results

A total of 993 APPs were initially identified in the iOS and Android platforms. After reading the titles and descriptions of the apps, 760 were excluded, leaving 233 eligible for review. After the apps were downloaded and filtered by criteria, 56 met the criteria for data extraction, 26 of which were available on Android and 30 were available on iOS. The complete process is illustrated in Figure 1 .

**Figure 1:** – Application selection process.

Characteristics of the apps are summarized in Table 1 .

**Table 1:** – Summary of the tools found in the applications

Variable	Total (n=56) n (%)	Android (n=26) n (%)	iOS (n=30) n (%)	p-value
**Blood pressure**				
Recording of values	55 (98)	26 (100)	29 (97)	1.000
**Medicines**				
Medications used	16 (29)	9 (35)	7 (23)	0.388
Medication reminder	19 (34)	13 (50)	6 (20)	0.025
**Other resources**				
Recording of comorbidities	8 (14)	6 (23)	2 (7)	0.127
PA captured or reported	11 (20)	6 (23)	5 (17)	0.738
Health education	14 (25)	8 (31)	6 (20)	0.375
Possibility of exporting data	12 (21)	2 (8)	10 (33)	0.025
Forums	2 (4)	2 (8)	0 (0)	0.211
Partnership with healthcare organization	3 (5)	3 (12)	0 (0)	0.094

PA: physical activity.

The tool related to recording of BP values was present in 98% of APPs. Only 29% of apps requested registration of medications used, and only 34% provided reminders to the use of these medications, which was more common in Android APPs (50%) than iOS (20%) (p=0.025). The option to include comorbid conditions was present in 14.0% of the apps. Only 20% of the analyzed apps had the possibility to capture or report PAs and 25% offered health education content for SAH control. Furthermore, 21% of APPs had the functionality to export data, being more frequent in iOS APPs (33%) compared to Android (8%) (p=0.025). Only 4% had discussion forums and 5% had a partnership with a health organization.

### Application quality

The average score of the quality of the APPs was 3.2±0.69. The highest score was for the functionality of the devices (3.7±0.73), while information analysis received the lowest average (3.0±0.71). When comparing the scores of Android apps with iOS apps, there was a statistically significant difference in terms of information (p=0.001), with Android apps being better evaluated (3.3±0.70 vs 2.7±0.60) ( Table 2 ).

**Table 2:** – Average application quality using the Mobile App Rating Scale by Stoyanov1

MARS subscales	Total	Android	iOS	p-value
Engagement	3.1 (.72)	3.2 (.74)	3.0 (.69)	0.211
Functionality	3.7 (.73)	3.8 (.86)	3.5 (.58)	0.107
Aesthetics	3.2 (.75)	3.3 (.79)	3.1 (.72)	0.361
Information	3.0 (.71)	3.3 (.70)	2.7 (.60)	0.001
Overall MARS score*	3.2 (.69)	3.4 (.74)	3.1 (.61)	0.080

MARS: Mobile App Rating Scale; data presented in mean (standard deviation). *Average of the four objective subscales.

### iOS apps with the highest averages

Table 3 presents the top five apps available in the App Store according to the MARS.

**Table 3:** – The top five iOS apps rated by Mobile App Rating Scale (MARS)

Characteristics	iOS apps				
	Mappa	AMPA	Blood Pressure Calendar	Blood Pressure Control	Elfie
**MARS score**	3.7	3.9	3.9	4.0	4.5
**Personal data**					
**Blood pressure**					
Self-recording of values		x	x	x	x
**Medicines**					
Medicines used		x	x		x
Reminder to use			x		
**Other resources**					
Self-recording of comorbidities		x		x	
PA captured or reported		x	x		x
Health education	x			x	x
Possibility of exporting recorded data		x	x		
Forums				x	
Partnership with healthcare organization				x	

PA: physical activity.

All APPs on the iOS platform with the highest MARS scores had a tool related to self-recording of BP values. Of the five APPs, three had some way of measuring the amount of PA in the individual with SAH.

### Android apps with the highest MARS averages

Table 4 presents the top five apps available in the Android app store according to the MARS.

**Table 4:** – The five highest rated Android applications by Mobile App Rating Scale (MARS)

Characteristics	Android apps				
	Blood pressure - Blood journal	Heart monitor: blood pressure diary	Blood pressure (bpresso)	UCS Digital Health	Blood Pressure Diary
**MARS score**	4.0	4.0	4.0	4.1	4.5
**Personal data**					
**Blood pressure**					
Register of values	x	x	x	x	x
**Medicines**					
Medicines used			x		
Warning for use		x	x		
**Other resources**					
Register of comorbidity		x	x	x	
PA captured or reported			x	x	
Health education		x			
Transformation into DM					
Forums					
Partnership with healthcare organization					

PA: physical activity; MARS: Mobile App Rating Scale.

All five best-rated Android apps also had a BP log for self-recording of values. Only two of the apps presented some way of measuring the amount of PA in the individual with SAH. The complete list of APPs evaluated with their respective overall scores on the MARS scale are available as supplementary material. Furthermore, in the 2024 update, it was observed that nine apps were discontinued, four in the Android store and five in the iOS store (highlighted in the supplementary material).

## Discussion

The objective of the study was to identify and analyze mobile APPs aimed at controlling BP, available in Portuguese on the two main platforms (App Store and Google Play). Among the main findings, it is highlighted that 55 apps (98%) have the option of recording BP values, while the tool for adherence to medication use was present only in 16 apps (29%), and medication reminders in 19 apps (34%). The relationship between BP self-management and PA practice did not reach ¼ of the apps. The overall mean MARS score was 3.2±0.69, with information, engagement, aesthetics and functionality in ascending order of scores on the MARS scale.

BP self-monitoring seems to indicate better control of BP values^14^and to be associated with greater medication adherence.^15^However, few APPs (21%) offered the possibility to export BP values and the option of generating reports with graphs and tables, making communication between doctor and patient difficult. In a previous study, around half of the apps (44%) analyzed had the option to export the data filled in by participants, facilitating the sharing of data with health professionals.^16^Another study reported that the main functionality of the apps analyzed was the educational content provided about the disease (59.1%), and secondly the option of self-monitoring of the disease (53.2%).^17^However, the authors reported that the educational contents were not checked to analyze the quality of the information.

Although the low adherence to pharmacological treatment by hypertensive patients is already well established,^3 , 18 , 19^the apps analyzed do little to explore tools to increase adherence to drug treatment. Meta-analyses showed that APPs with tools aimed at controlling pharmacological treatment tend to increase adherence to the use of prescribed medications.^8 , 20^Therefore, apps must have a system for issuing notifications, through prior filling out of medication times, aiming to remind users to take their medications at the prescribed times, facilitating medication adherence.^21 , 22^

Behavioral changes are fundamental for the control of BP, but we found that Brazilian apps barely address this issue in their functionalities. In the present study, only 14 (25%) apps presented tools aimed at health education for users, and 11 (20%) of the applications contained tools that facilitated adherence to PA. The promotion of behavioral changes supported by mobile health, or mHealth, in the hypertensive and diabetic population has been investigated in recent years, and appears to direct individuals towards healthier habits.^23 - 25^Even though more studies are needed to prove the effectiveness of features to increase positive behaviors, it is important that apps explore these variables to encourage preventive behaviors related to lifestyle for the non-pharmacological treatment of hypertensive individuals.^3^

In the context of a more qualitative assessment based on the MARS scale, the functionality variable was the best evaluated (3.7±0.73 out of 5). Comparisons between the scores obtained by the Android and iOS apps showed that, in all subscales, the apps available in the Android obtained a slight advantage, with a statistically significant difference (p=0.001) only for the information aspect. We can observe similarities with previous studies, in which the objective category with the highest MARS score was functionality, with an overall average of 3.5 out of 5.0. However, unlike the present study, a study^11^assessing apps available in the Dutch official app stores for Android and iOS platforms reported similar overall MARS score was found in Android and iOS apps (2.63 vs 2.64).

It is also worth mentioning that the information resources of the apps included in this study were often missing or of low quality. Other studies also reported low quality of the information resource present in apps.^11 , 16 , 26 , 27^This low quality and/or lack of information may result in incorrect use of the app, since many users unfamiliar with the app may enter and interpret data in the wrong way. Therefore, it is important that apps focus on simple interfaces, attractive design, clear and objective texts, to facilitate its use.^28^

To the best of our knowledge, only one study^29^was published evaluating apps focusing on BP control, available in Portuguese language. This^29^was a narrative review, where the authors carried out a search only on the Google Play Store, including a total of 267 APPs. In this mentioned study, the authors listed the apps by category, number and main functionalities, and highlighted that most apps were classified as entertainment, with functionalities that did not have a scientific character, underscoring the importance of better control in relation to the development and availability of mHealth apps.^29^The present study therefore offers pertinent information regarding the quality, functionality, aesthetics, engagement and information of apps available in Portuguese. Furthermore, our study describes differences between iOS and Android apps.

This study has limitations, such as not including paid APPs or analyzing only simpler versions, which limits our conclusions. Since this is a cross-sectional study, changes in the trends of app functionalities are disregarded. Furthermore, the fact that duplicate apps in both stores (iOS and Android) were analyzed in a single platform and in a random manner should be considered in the interpretation of our findings.

In the present study, we identified the number of apps on BP control available in Portuguese in both Android and iOS platforms. Most apps were of acceptable quality, but, in general, they were not comprehensive in terms of the factors related to BP control. There is no study assessing the effectiveness of these apps in controlling BP, highlighting the need for the development of studies for this purpose. Also, our findings emphasize the importance of developing apps that promote greater pharmacological and non-pharmacological treatment adherence, and reliability for health professionals to recommend their use, given that little knowledge about apps is a barrier to their recommendation.^28^
